# Water Extract from the Leaves of *Withania somnifera* Protect RA Differentiated C6 and IMR-32 Cells against Glutamate-Induced Excitotoxicity

**DOI:** 10.1371/journal.pone.0037080

**Published:** 2012-05-14

**Authors:** Hardeep Kataria, Renu Wadhwa, Sunil C. Kaul, Gurcharan Kaur

**Affiliations:** 1 Department of Biotechnology, Guru Nanak Dev University, Amritsar, India; 2 National Institute of Advanced Industrial Science and Technology, Tsukuba, Japan; Universidade Federal do Rio de Janeiro, Brazil

## Abstract

Glutamate neurotoxicity has been implicated in stroke, head trauma, multiple sclerosis and neurodegenerative disorders. Search for herbal remedies that may possibly act as therapeutic agents is an active area of research to combat these diseases. The present study was designed to investigate the neuroprotective role of *Withania somnifera* (Ashwagandha), also known as Indian ginseng, against glutamate induced toxicity in the retinoic acid differentiated rat glioma (C6) and human neuroblastoma (IMR-32) cells. The neuroprotective activity of the Ashwagandha leaves derived water extract (ASH-WEX) was evaluated. Cell viability and the expression of glial and neuronal cell differentiation markers was examined in glutamate challenged differentiated cells with and without the presence of ASH-WEX. We demonstrate that RA-differentiated C6 and IMR-32 cells, when exposed to glutamate, undergo loss of neural network and cell death that was accompanied by increase in the stress protein HSP70. ASH-WEX pre-treatment inhibited glutamate-induced cell death and was able to revert glutamate-induced changes in HSP70 to a large extent. Furthermore, the analysis on the neuronal plasticity marker NCAM (Neural cell adhesion molecule) and its polysialylated form, PSA-NCAM revealed that ASH-WEX has therapeutic potential for prevention of neurodegeneration associated with glutamate-induced excitotoxicty.

## Introduction

Research into medicinal plants so as to identify the novel, natural and safe phytotherapies has flourished and recently several *in vitro* and *in vivo* pre-clinical studies validating the therapeutical value of newly identified phytochemicals have been launched. Presently, many of the traditional herbal medicines are increasingly being appreciated with Western models of integrative health sciences and evidence-based approach both in research and clinic [1]. In contrast to the conventional single-module medicine, the herbal extracts function through multi-target mechanisms and hence may hold key to the success where conventional agents fail [2]. Brain pathologies pose an extra degree of complexity in their treatment and hence there is a compelling reason to search for naturotherapeutic ways. Recently, many studies have focused on the potential of crude extracts and their isolated compounds from fruits, vegetables and herbs to prevent certain neurological disorders. Some beneficial phytochemicals from *Curcuma longa*, *Withania somnifera*, *Panax ginseng*, *and Ginkgo bilobae etc.* [3], [4], [5], [6] have been identified that exhibit significant neuroprotective effects in various experimental models of neurological disorders. The proposed underlying mechanisms include preconditioning, antioxidation and anti-inflammation effects. Some of the herbs have been classified as brain tonics or rejuvenators in Ayurveda, the traditional Indian medicine system. Among these, the most important plant is Ashwagandha whose extracts make a significant component to the daily supplements for body and brain health. Although a variety of Ashwagandha extracts have displayed neuroprotective, neuroregenerative and anticancer potentials in recent *in vitro* studies [7], [8], [9], [10], [11] using brain-derived cells, potentials of water extract of leaves of Ashwagandha (ASH-WEX) remain largely unexplored. In the present study, we used glutamate induced excitotoxicity as a model to investigate the neuroprotective potentials of ASH-WEX.

Glutamate is the major excitatory neurotransmitter in the CNS where it acts upon ionotropic (N-methyl-D-aspartate (NMDA) and α-amino-3-hyroxy-5-methylisoxazole proprionic acid (AMPA)) or metabotropic (mGlu1-mGlu8) receptors [12], [13]. Although glutamate plays a central role in excitatory neurotransmission, alterations in glutamate homeostasis can have significant repercussions on neural cells through the generation of neurotoxic or excitotoxic cascades [14], [15]. Abnormalities in glutamate neurotransmitter system are not only involved in acute neural trauma such as ischemia, spinal cord injury, head trauma, and epilepsy, but also in neurodegenerative disorders such as Huntington's, Alzheimer's and Parkinson's diseases, amyotrophic lateral sclerosis, AIDS complex, and domoic acid neurotoxicity [16], [17], [18]. After brain ischemia or traumatic injury to the CNS, there is a pathological release of glutamate from neurons and glial cells [19], [20]. Glutamate uptake by astrocytes normally prevents excitotoxic glutamate elevations in brain extracellular space [21]. The uncontrolled release of glutamate can lead to a constant stimulation of glutamate receptors and the deregulation of intracellular Ca^++^ homeostasis, mainly through NMDA receptor activation. However, in an excitatory crisis, the potentially protective functions of reactive astrocytes, such as glutamate uptake and elimination of free radicals can eventually be reduced or even reversed and might instead contribute to the development of neural damage [22], [23]. Thus, activated astrocytes might both protect from and contribute to the glutamate-mediated neuronal damage. As glutamate neurotoxicity is involved in the pathogenesis of various diseases, reduction of glutamate toxicity is one of the important therapeutic strategy for drug designig [24], [25], [26] and several drugs targeting glutamate toxicity are under development.

**Table 1:** Primer sequences used for semi-quantitative RT-PCR.

No.	mRNA	Primer Sequence	Expected product size
1.	GFAP	F 5′GGCGCTCAATGCTGGCTTCA3′R 5′TCTGCCTCCAGCCTCAGGTT3′	326 bp
2.	NF200	F 5′CAAGGAACCCAGCAAACCA3′R 5′GGCCTCTGTCTTGGGTTTCTC3′	106 bp
3.	HSP70	F 5′GAGTTCAAGCGCAAACACAA3′R 5′CTCAGACTTGTCGCCAATGA3′	428 bp
4.	NCAM	F 5′TGAGGGTACTTACCGCTGTG3′R 5′GTTGCTGGCAGTGCACATGT3′	651 bp
5.	PST	F 5′TAAGGTGCAATCTAGCTCCTGTGGTGG3′R 5′GCATCCTGTGAGGACTGGCGTTGGAAA3′	474 bp
6.	β-actin	F 5′TCACCCACACTGTGCCCATCTACGA3′R 5′CAGCGGAACCGCTCATTGCCAATGG3′	285 bp

The molecular mechanisms of cell death induced by glutamate have not been fully elucidated [27]. Since the free radical-scavenging agents and antioxidants such as, vitamin E [28] are shown to have protective impact on glutamate-toxicity, excessive accumulation of free radicals has been speculated to be responsible, at least in part, for glutamate-induced neuropathologies. Curcumin (a major componet of turmeric) and epicatechin-gallate (a major component of green tea) have been shown to protect primary cultured neurons from glutamate-induced cell death [29].

**Figure 1:**
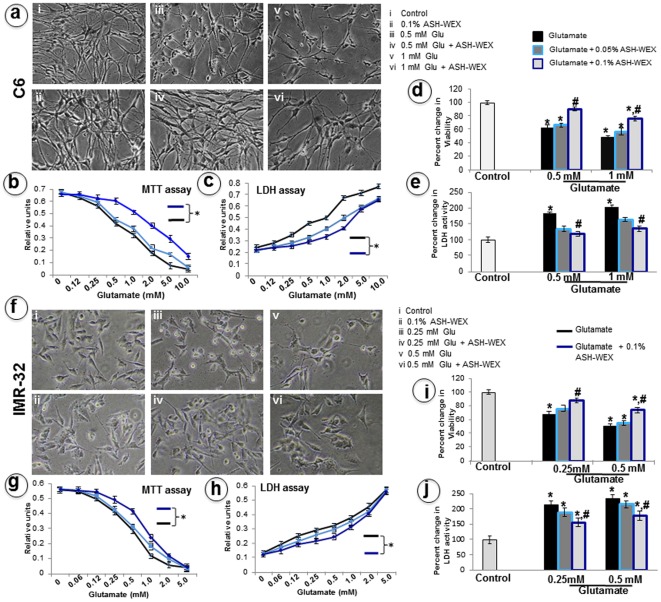
The morphological changes in C6 (a) and IMR-32 (f) cells were studied using phase contrast images. Cell viability and toxicity of various concentrations of glutamate was assayed by MTT and LDH assays in RA differentiated C6 (b,c) and IMR-32 (g,h) cells. (d) and (i) histograms represents the relative percentage viability of glutamate and ASH-WEX treated C6 and IMR-32 cells, respectively, as compared to the control cells. (e) and (j) histograms represents the relative LDH activity when the control and ASH-WEX pretreated cells were exposed to different glutamate concentrations. “*” represents the statistical significant difference between all the treatment groups (glutamate alone or glutamate + ASH-WEX groups) with respect to control group. “#” represents the statistical difference between “glutamate + ASH-WEX” treated groups with their respective “glutamate” treatment groups. “*” and “#” = p<0.05.

**Figure 2:**
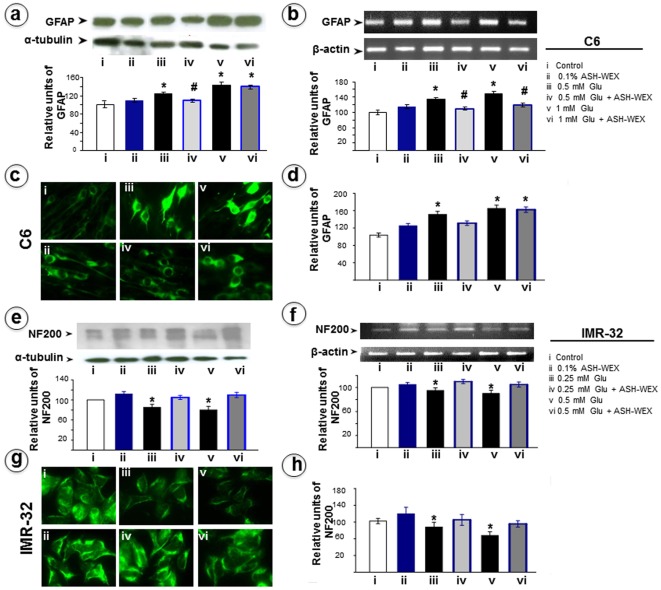
Representative Western blots and their densitometry analysis for GFAP (a) and NF200 (e) for RA differentiated C6 and IMR-32 cells, respectively. RT-PCR results for GFAP and NF200 mRNA in C6 (b) and IMR-32 (f) cells, respectively and their relative densitometry analysis was represented by histograms. The expression of GFAP in C6 (c) and NF200 in IMR-32 (g) cells was analysed by immunocytostaining and relative intensity was plotted as histogram as analysed by Image pro-plus software. “*” represents the statistical significant difference between all the treatment groups (ASH-WEX alone, glutamate alone or glutamate + ASH-WEX groups) with respect to control group. “#” represents the statistical difference between “glutamate + ASH-WEX” treated groups with their respective “glutamate” treatment groups. “*” and “#” = p<0.05.

**Figure 3:**
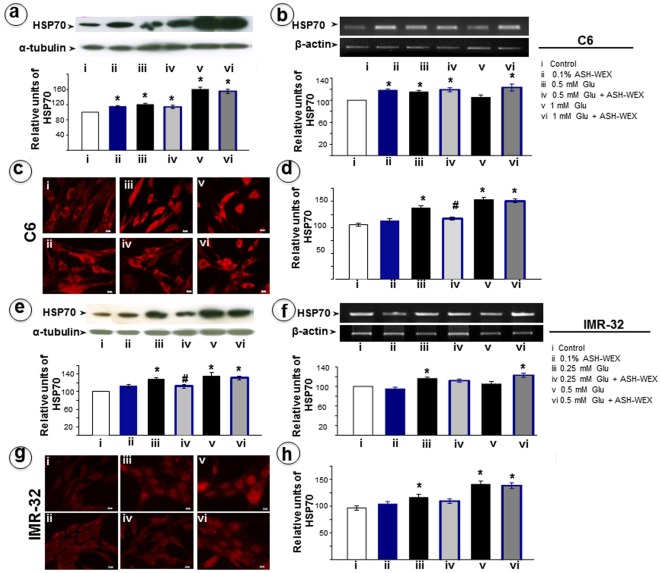
Representative Western blots and their densitometry analysis for HSP70 in RA differentiated C6 (a) and IMR-32 (e) cells, respectively. RT-PCR results for HSP70 mRNA in C6 (b) and IMR-32 (f) cells, respectively and their relative densitometry analysis was represented by histograms. The expression of HSP70 in C6 (c) and IMR-32 (g) cells was analysed by immunocytostaining and relative intensity was plotted as histogram as analysed by Image pro-plus software. “*” represents the statistical significant difference between all the treatment groups (ASH-WEX alone, glutamate alone or glutamate + ASH-WEX groups) with respect to control group. “#” represents the statistical difference between “glutamate + ASH-WEX” treated groups with their respective “glutamate” treatment groups. “*” and “#” = p<0.05.

**Figure 4:**
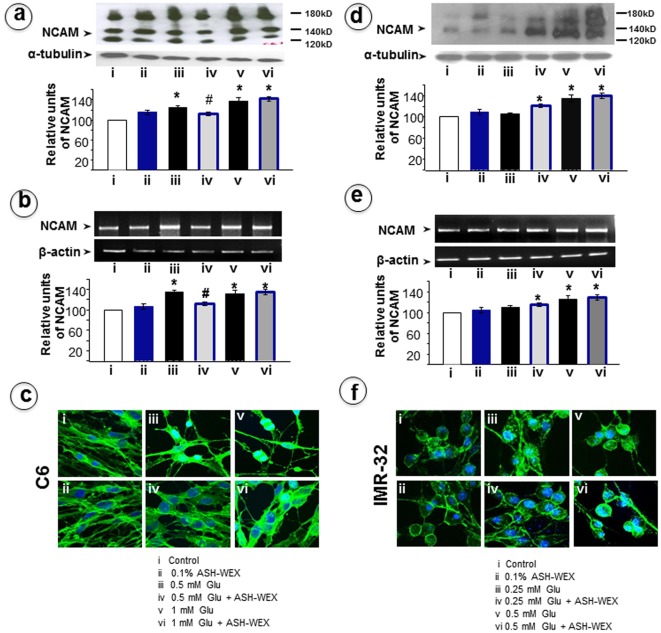
Representative Western blots and their densitometry analysis for NCAM in RA differentiated C6 (a) and IMR-32 (d) cells, respectively. RT-PCR results for NCAM mRNA in C6 (b) and IMR-32 (e) cells, respectively and their relative densometery analysis was represented by histograms. The expression of NCAM in C6 (c) and IMR-32 (f) cells was analysed by immunostaining. “*” represents the statistical significant difference between all the treatment groups (ASH-WEX alone, glutamate alone or glutamate + ASH-WEX groups) with respect to control group. “#” represents the statistical difference between “glutamate + ASH-WEX” treated groups with their respective “glutamate” treatment groups. “*” and “#” = p<0.05.

**Figure 5:**
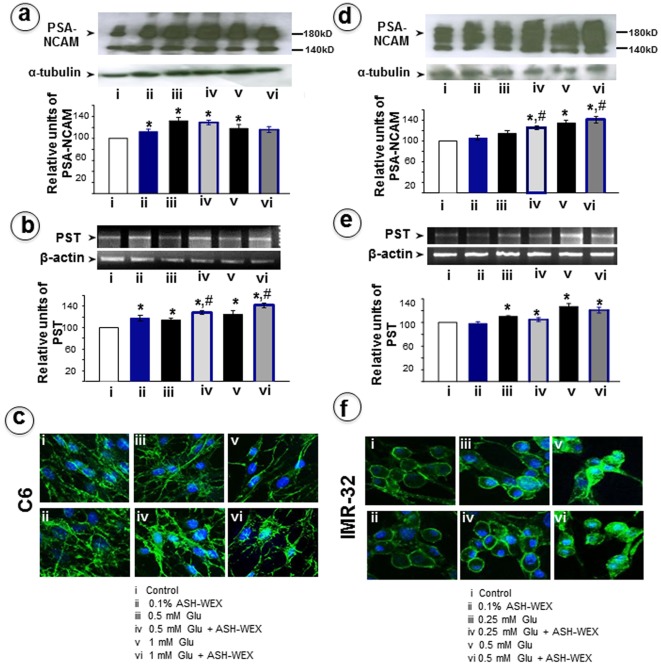
Representative Western blots and their densometery analysis for PSA-NCAM in RA differentiated C6 (a) and IMR-32 (d) cells, respectively. RT-PCR results for PST mRNA in C6 (b) and IMR-32 (e) cells, respectively and their relative densometery analysis was represented by histograms. The expression of PSA-NCAM in C6 (c) and IMR-32 (f) cells was analysed by immunostaining. “*” represents the statistical significant difference between all the treatment groups (ASH-WEX alone, glutamate alone or glutamate + ASH-WEX groups) with respect to control group. “#” represents the statistical difference between “glutamate + ASH-WEX” treated groups with their respective “glutamate” treatment groups. “*” and “#” = p<0.05.

**Figure 6:**
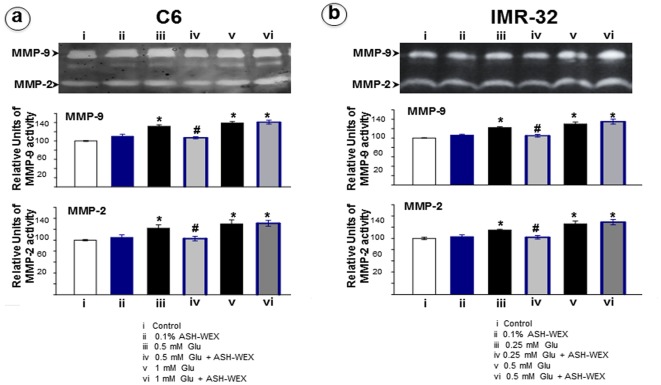
Representative Gelatin Zymograms for MMP 2 & 9 from media obtained from different groups of C6 (a) and IMR-32 (b) cells. The zymograms were analysed using spot –denso method in Alpha Ease software and data was represented as histograms. “*” represents the statistical significant difference between all the treatment groups (ASH-WEX alone, glutamate alone or glutamate + ASH-WEX groups) with respect to control group. “#” represents the statistical difference between “glutamate + ASH-WEX” treated groups with their respective “glutamate” treatment groups. “*” and “#” = p<0.05.

The present study was designed to test the hypothesis that Ashwagandha leaves derived water extract (ASH-WEX) may confer protection against glutamate induced toxicity. Retinoic acid (RA) differentiated C6 glioma and IMR32 neuroblastoma cells have been widely accepted for *in vitro* studies due to their close resemblance to glial and neuronal cells, respectively [30], [31]. Expression analysis of glial and neuronal cell markers (glial fibrillary acidic protein (GFAP) and Neurofilament 200 (NF200), respectively) was made following treatments with glutamate and ASH-WEX. Upregulation of GFAP is a marker for reactive gliosis, trauma and degeneration in CNS whereas; HSP 70 is a useful stress response marker. Experiments using both animal models of stroke and tissue culture systems have indicated that overexpression of HSP70 reduced ischemic injury and protected both neurons and glial cells [32], [33]. Further the expression of stress related marker HSP70 along with plasticity markers NCAM and PSA-NCAM was evaluated to establish their role in ASH-WEX mediated neuroprotection. NCAM, a member of the immunoglobulin superfamily of cell recognition molecules, is widely expressed on axons and dendrites [34] and is a key regulator of neuronal development and function [35]. NCAM is associated with an unusual glycan, polysialic acid, a highly negatively charged and voluminous carbohydrate modulating its adhesive and concomitant signal transduction functions. NCAM and PSA-NCAM play important roles in the development of the nervous system and NMDA receptor dependent synaptic plasticity in the adult [36]. The induction of PSA expression in damaged adult CNS tissues has been shown to be promising therapeutic target in repair, remodelling and regeneration [37]. Use of crude leaf water extract is both eco- and bio-friendly as neither there is a need to sacrifice whole plant (as in case of roots) nor any organic solvents are required. Moreover the aqueous extract is easy to prepare and convenient as well as safe to use.

## Materials and Methods

### Preparation of the water extract of leaves of Ashwagandha (ASH-WEX)

ASH-WEX was prepared as reported earlier [38]. Briefly, 10 g of dry leaf Ashwagandha powder was suspended in 100 ml of sterile distilled water. It was incubated at 45°C for overnight with slow stirring. The slurry was centrifuged at 10,000 rpm and was then filtered under sterile conditions. The filtrate so obtained was treated as 100% ASH-WEX.

### Cell culture and treatments

C6 (rat glioma) and IMR-32 (human neuroblastoma) cells were purchased from National Centre for Cell Science (Pune, India). The cells were routinely grown in DMEM supplemented with 10% Fetal bovine serum and 1× PSN mix (Invitorgen) at 37°C in a humidified atmosphere containing 5% CO_2_.

Undifferentiated cultures were subcultured by trypsinization and cultured in 96 and 24 well plates according to the requirement of the experiment. After 24 hrs of seeding, C6 and IMR-32 cells were differentiated for 4 and 6 days, respectively by adding retinoic acid (RA) to the culture medium to a final concentration of 10 µM. The medium was changed every two days. RA differentiated cultures were pretreated with 0.05% and 0.1% ASH-WEX for 24 hrs and then exposed to glutamate (0.06 mM–10 mM) in the presence of ASH-WEX.

### Proliferation and Cytotoxicity assays

ASH-WEX was tested for protective activity against glutamate on C6 and IMR-32 cells using the 3-(4, 5-dimethylthiazol-2-yl)-2, 5-diphenyltetrazolium bromide (MTT) test. This method is based on the reduction of the tetrazolium salt MTT into a crystalline blue formazan product by the cellular oxidoreductase. The amount of formazan produced is considered as a relaible representation of viable cell number. After 24 hrs of treatment with glutamate, the culture medium was removed and replaced with fresh culture medium containing MTT (0.5 mg/ml). After 4 h incubation at 37°C, this solution was removed, and the resulting blue formazan was solubilized in 100 µl of DMSO and the optical density was read at 595 nm using microplate reader (Multiskan PLUS, Thermo Scientific).

In order to assay the cytotoxicity, Lactate dehydrogenase (LDH) assay was done as described by Abe and Matsuki [39]. Briefly, LDH substrate mixture (1 ml) was prepared as follows; 2.5 mg l-lactate lithium salt and 2.5 mg NAD were dissolved in 0.9 ml of 0.2 M Tris–HCl buffer (pH 8.2) with 0.1% (v/v) Triton X-100, and 0.1 ml of MTT stock solution (2.5 mg/ml) and 1 µl of MPMS stock solution (100 mM) were added. 50 µl of the culture supernatant was transferred to 96-well culture plates, and mixed with 50 µl of the LDH substrate mixture. The reaction was stopped by adding 100 µl of a solution containing 50% dimethylformamide and 20% sodium dodecyl sulfate (DMF/SDS, pH 4.7). The absorbance was measured at 570 nm with Multiskan PLUS reader (Thermo Scientific).

### Immunostaining

All cells, control and treated, were fixed with 4% paraformaldehyde followed by permeabilization with 0.3% Triton X-100 in Phosphate buffer saline (PBST). Cells were incubated with anti-GFAP (1∶500, Sigma), anti-HSP70 (1∶1000, Sigma), anti NF200 (1∶500, Sigma), anti-NCAM (1∶500, Sigma) and anti PSA-NCAM (1∶250, AbCys) diluted in 0.1% PBST, for 24 h at 4°C in humid chamber. Secondary antibody (Alexa Fluor 488, 546, Invitrogen) was applied for 2 h at room temperature. Cells were incubated with (DAPI, 1∶5000 in 0.1%PBST) for 10 minutes for nuclear staining and then mounted with anti-fading reagent (Fluoromount, Sigma) and observed under the microscope (Nikon A1RConfocal). Images were captured under (40×) and were analyzed using image pro-plus software version 4.5.1 from the media cybernetics.

### Protein assay and Western blotting

Cells grown and treated in 10 cm dishes were harvested with PBS-EDTA (1 mM). Cell pellet was lysed in homogenizing buffer (50 mM Tris, 150 mM NaCl, 1 mM EDTA, 100 µM NaVO4, 1 mM PMSF and 0.5 mM DTT, Protease Inhibitor cocktail) and protein content in the supernatant was determined by the Bradford method.

Cell lysate (20–30 µg) was resolved on 8–10% SDS-PAGE followed by transfer onto a PVDF membrane (Hybond-P) using the semidry Novablot system (Amersham Pharmacia). Subsequently, membranes were probed with mouse anti-GFAP (1∶3000), anti-HSP70 (1∶1000),Anti-NF200 (1∶3000), anti-NCAM (1∶2500) or anti-PSA-NCAM (1∶2000) antibody. Immunoreactive bands were visualized using ECL Plus Western blot detection system (Amersham Biosciences). In order to account for potential variations in protein estimation and sample loading, expression of each protein was compared to that of α-tubulin.

### Semi-quantitative RT-PCR

Total RNA was extracted from cells by the TRI reagent (Sigma) according to manufacturer's instruction. Equal amounts of RNA were used for cDNA synthesis in 20 µl reactions containing 200 U M-MLV reverse transcriptase, 4 µl 5×first strand buffer (Fermentas), 5 µg of total RNA, 1 mM each of dNTPs (Fermentas), 20 units of ribonuclease inhibitor (Sigma), and 250 ng pd(N)_6_ random hexamers (Fermentas). 2 µl of cDNA was amplified in a 50 µl PCR reaction mixture containing two units Taq polymerase, 5 µl 10× PCR buffer, 3.0 µl of 25 mM MgCl_2_ (Sigma), 1 µl of 10 mM dNTP mix (Fermentas), and 20 picomoles of respective primers as listed in Table 1. Cycling conditions comprised of an initial denaturation of 3 min at 94°C followed by 35 cycles of amplification (at 94°C for 40 sec, 55°C for 45 sec and 72°C for 1 min) and final elongation step at 72°C for 10 min. To control the PCR reaction components and the integrity of the RNA, 2 µl of each cDNA sample was amplified separately for β-actin specific primers.

### Gelatinase Zymography

Gelatinase Zymography is simple, sensitive, quantifiable, and functional assays to analyze MMPs in biological samples which identifies MMPs by the degradation of their preferential substrate gelatine and by their molecular weight. Gelatin zymography is mainly used for the detection of the gelatinases, MMP-2 and MMP-9 as gelatin is the specific substrate for these two MMPs. Gelatinase zymography was performed in 10% SDS Polyacrylamide Gel in the presence of 0.1% gelatin under nonreducing conditions. Culture media (20 µl) were mixed with sample buffer and loaded for SDS-PAGE. Samples were not boiled before electrophoresis. Following electrophoresis the gels were washed twice in 1× Renaturing Buffer (Invitrogen) for 30 min at room temperature to remove SDS. The gels were then incubated at 37°C for 48–72 hrs in Developing Buffer (Invitrogen) stained with 0.5% Coomassie Blue R250 in 50% methanol and 10% glacial acetic acid for 30 min and destained. Upon renaturation of the enzyme, the gelatinases digest the gelatin in the gel and give clear bands against an intensely stained background.

### Data Analysis

The captured images were analyzed using Image Pro-Plus software version 4.5.1 from Media Cybernetics. The extent of GFAP, NF 200, HSP70 immunoreactivity was quantified by the overall density of their respective immunoreactivity each in 5–6 randomly selected fields on each image using the count/size command of the Image Pro-Plus software. 15 different images were used from three different experiments and the data were averaged and expressed as percentage with respect to control.

### Statistical Analysis

Data was analyzed statistically using Sigma Stat for Windows (version 3.5). The results were analyzed using One-way ANOVA to determine the significance of the mean between the groups. Values of p≤0.05 were considered significant. The means of the data are presented together with the standard error mean (SEM).

## Results

### ASH-WEX attenuated glutamate-induced cytotoxicity

To determine whether glutamate can induce the death of RA differentiated C6 and IMR-32 cells, differentiated cultures were treated with various doses of glutamate (0.06 mM–10 mM). Cultures treated with glutamate concentrations (0.5 mM and 1 mM for C6 cells and 0.25 mM and 0.5 mM for IMR-32 cells) for 24 h exhibited cell shrinkage and rounding (Fig. 1a-iii,v and 1f-iii,v). MTT assay on glutamate treated cells revealed a decrease of the number of living cells after treatment with increasing doses of glutamate (Fig. 1b, g). The extent of glutamate toxicity for RA-differentiated C6 glial and IMR-32 neuronal cells was different; whereas 1 mM glutamate exposure induced more than 50% C6 glial cell death, IMR-32 neuronal cells showed similar effect with 0.5 mM glutamate. Using these doses as toxicity models, we next investigated whether ASH-WEX (0.05% and 0.1%) could protect differentiated C6 and IMR-32 cells against glutamate-induced cell death. Pretreatment for 24 hrs with ASH-WEX (0.1%) significantly inhibited the death of C6 (Fig. 1b, d) and IMR-32 (Fig. 1g, i) cells exposed to glutamate. Glutamate-induced changes in the cell morphology were partially suppressed by treatment with 0.1% ASH-WEX. Of note, the recovery in the cell morphology was observed only for the low dose (0.5 mM for C6 and 0.25 mM for IMR-32) of glutamate treatment (Fig. 1a-iv, f-iv). LDH assay also confirmed the protective effect of ASH-WEX as the enzyme activity increased with rise in glutamate dose, implicating glutamate toxicity, which was significantly reduced in 0.1% ASH-WEX pretreatment group in C6 (Fig. 1c, e) and IMR-32 (Fig. 1h, j) cells. Based upon MTT and LDH assay a low (0.5 mM for C6, 0.25 mM for IMR-32) and high dose (1 mM for C6 and 0.5 mM for IMR-32) of glutamate was selected for further experiments.

### ASH-WEX abolished glutamate induced changes in the C6 and IMR-32 marker proteins

GFAP is an astrocyte-specific intermediate filament thought to provide structural support to normal astrocytes. Increase in GFAP production is a sign of astrogliosis, reactive injury, and neurodegeneration in the differentiated cells. Cells were exposed to 0.5 mM or 1 mM glutamate (24 h) after the pretreatment with 0.1% ASH-WEX (24 h). As shown in Fig. 2a, there was a significant increase (p<0.05) in GFAP expression in the C6 cells upon treatment with glutamate which was suppressed with ASH-WEX pre-treatment in the 0.5 mM glutamate treatment group. In the 1 mM glutamate treatment group 0.1% ASH-WEX was not able to normalize the GFAP expression level (Fig. 2a). We performed RT-PCR in the control and treated groups and found that there was a significant increase in GFAP mRNA in glutamate treated groups. ASH-WEX pre-treatment was able to suppress the upregulation in GFAP mRNA in both low and high dose glutamate groups (Fig. 2b). Single cell quantitative immunocytofluoroscence for GFAP in these groups further revealed dose dependent increase in GFAP expression in the glutamate-exposed as compared to the control cells (Fig. 2c and d). In cells pretreated with ASH-WEX, the expression level of GFAP was normalized in low dose glutamate group but there was no significant difference in expression of GFAP in high dose group (Fig. 2d).

Neurofilaments (NFs) belong to the family of intermediate filaments (IFs) and are structural elements of the neuronal cytoskeleton in an interconnection with actin microfilaments, microtubules and other IFs. NF-H (NF200) is expressed mainly in the differentiated neurons while other two forms are abundant in pre-natal stages. NF200 expression in IMR-32 cells was reduced upon glutamate exposure, whereas, pretreatment with ASH-WEX lead to recovery of glutamate induced decrease in NF200 levels shown by Western blotting (Fig. 2e). These results were supported by RT-PCR that revealed that ASH-WEX pretreatment resulted in recovery of NF200 mRNA in glutamate treated cells. There was 20–35% increase in NF200 mRNA expression in ASH-WEX pretreated groups as compared to their respective 0.25 mM and 0.5 mM glutamate groups (Fig. 2f). NF200 immunostaining confirmed the changes observed in Western blotting and RT-PCR results at single cell level (Fig. 2g) that was further quantitated by intensity analysis (Fig. 2h).

### ASH-WEX abolished glutamate-induced increase in HSP70

HSP70 is a member of heat shock protein family and serves as a housekeeper in the cell, assisting in the correct folding, trafficking, and degradation of many proteins during normal and stressed conditions. We examined HSP70 expression in control and ASH-WEX pretreated cells that were challenged with glutamate. As shown in Fig. 3a and e, HSP70 Western blots revealed significant increase after exposure to glutamate in a dose dependent manner in both C6 and IMR-32 cells suggesting that glutamate treatment evoked stress response in these cells. ASH-WEX treatment both in C6 and IMR-32 cells resulted in a moderate induction of HSP70 expression and led to normalization of increase in HSP70 induced by low dose of glutamate (Fig. 3a, e). Upregulation in HSP70 expression (about 60% increase in C6 cells and 40% in IMR-32 cells) induced by higher dose of glutamate was not recovered significantly upon ASH-WEX pre-treatment (Fig. 3a,e). The RT-PCR analysis showed increase (p<0.05) in HSP70 mRNA levels at low dose glutamate treatment group in both cell types; the high dose glutamate did not cause higher induction of HSP70 mRNA (Fig. 3b,f). Furthermore, the immunocytostaining for HSP70 showed enhanced intensity in glutamate treated groups as compared to control (Fig. 3c,g). ASH-WEX pre-treatment resulted in downregulation of HSP70 in low dose glutamate group in both the cell lines; high dose glutamate groups remained unaffected (Fig. 3d,h).

### ASH-WEX induces NCAM and PSA-NCAM expression to reduce excitotoxic cell death in glutamate challenged cells

NCAM is a glycoprotein of immunoglobulin (Ig) superfamily, expressed on the surface of neurons and glia cells. It has a role in cell–cell adhesion, neurite outgrowth, synaptic plasticity, neuroprotection and learning and memory. We examined NCAM expression in control and treated groups and found that ASH-WEX treatment caused a minor increase in NCAM expression both in C6 and IMR-32 cells (Fig. 4 a, d). The low dose treatment of glutamate (0.5 mM) led to upregulation of NCAM expression (p<0.05) which was further increased in the high dose glutamate (1 mM) treated cells as seen on the Western blots. ASH-WEX (0.1%) pre-treatment led to normalization of NCAM expression in the low dose glutamate group but its expression remained significantly higher (around 45%) in the high dose treatment group (Fig. 4a,d). These changes were also apparent at mRNA level. Lower dose of glutamate (0.5 mM) exposure led to increase in NCAM mRNA level that was normalized by ASH-WEX in C6 cells (Fig. 4b). On the other hand high dose treatment group did not show normalization of NCAM mRNA in C6 cells when pretreated with ASH-WEX. Furthermore, ASH-WEX did not cause any recovery in NCAM mRNA expression in both low and high dose glutamate groups of IMR-32 (Fig. 4e). Immunocytostaining for NCAM was enhanced upon glutamate exposure in case of C6 cells as well as in IMR-32 cells (Fig. 4 c, f). ASH-WEX pre-treatment induced normalization was evident by NCAM staining. Consistent with the protein and mRNA expression data, NCAM immunocytostaining in IMR-32 cells revealed that ASH-WEX was not able to recover cells from glutamate-induced changes.

The polysialylated neuronal cell adhesion molecule (PSA-NCAM) is considered as a marker of developing and migrating neurons and of synaptogenesis in the immature vertebrate nervous system. However, it persists in the mature normal brain in some regions which retain a capability for morphofunctional reorganization throughout life. We examined PSA-NCAM in control and treated groups and found that glutamate exposure led to an increase in the PSA-NCAM expression by about 25% at low glutamate dose both in C6 and IMR-32 cells which was further enhanced in ASH-WEX pre-treatment group in the IMR-32 cells (Fig. 5 a,d). The PSA-NCAM was around 15% (p<0.05) higher at high dose glutamate treatment group in C6 cells as compared to control. ASH-WEX pretreated group did not show any significant change (Fig. 5a). In contrast, there was a dose dependent increase in PSA-NCAM expression in the IMR-32 cells from 15–45% that was further enhanced in the ASH-WEX pretreatment (Fig. 5d). The expression of polysialyltransferase (PST) mRNA was examined by RT-PCR and was found to be significantly increased both in glutamate and ASH-WEX treatment groups as compared to control (Fig. 5 b,e). Immunocytostaining revealed that PSA-NCAM expression was enriched along the projections of the differentiated cells in the control group that was further enhanced by low glutamate treatment both in the C6 and IMR-32 cells. High dose glutamate led to disruption of surface expression of PSA-NCAM both in C6 and IMR-32 cells (Fig. 5 c,f).

### ASH-WEX modulated MMP-2 and 9 expression after glutamate exposure

Matrix metalloproteinase (MMPs) are a family of proteinases that function to cleave virtually all components of the extracellular matrix (ECM), making them excellent mediators of early inflammatory processes, tissue remodeling and scar formation following a variety of injury types. In particular, the gelatinases, MMP-2 (gelatinase A) and MMP-9 (gelatinase B) degrade common ECM components, as well as the major CNS matrix component, chondroitin sulfate proteoglycans (CSPGs). MMP-2 and MMP-9 have been linked to blood–brain barrier disruption, inflammation, angiogenesis, remodeling of the ECM and glial scar formation and are associated with extracellular remodeling that occurs in injury and repair processes in the CNS. The expression and activity of MMP-2 and 9 was studied by gelatin zymography. The expression/activity of both these enzymes was increased in glutamate treatment groups as apparent by the area of the white bands. ASH-WEX reduced the enzyme activity significantly upon treatment in low dose glutamate exposed cultures but was unable to induce any significant changes in high dose glutamate group in both the cell lines (Fig. 6 a,b).

## Discussion

The current data reveal that the ASH-WEX ameliorated glutamate induced decrease of cell viability in a dose-dependent manner. Moreover, glutamate-induced apoptosis/necrosis was also attenuated after treatment with ASH-WEX as evident from LDH assay and phase contrast images of cells. Although Ashwagandha has been reported to improve learning and memory in rats and as a potent neuroprotectant [40], but the water soluble Ashwagandha leaf extract has not been evaluated for its cytoprotective effects.

In the present study, enhanced expression of GFAP upon glutamate exposure of RA differentiated C6 cells may be attributed to reactive gliosis and its induction. Upregulation of intermediate filament proteins, in particular GFAP by reactive astrocytes is perhaps the best known hallmark of reactive astrocytes and reactive gliosis. IF upregulation has been found in CNS trauma, hypoxia, around growing tumors, and in many neurodegenerative conditions [41]. Recently it has been demonstrated that a crosstalk between GFAP and glutamate signalling exists and the expression of GFAP is essential to anchor the glutamate transporter GLAST in the astrocyte plasma membrane thus enhancing GLAST-mediated transport [42]. Also GFAP knockout mice exhibit reduced glutamate clearance [43]. Thus changes in GFAP gene expression and glutamate homeostasis might mutually influence each other. Glutamate activates the GFAP gene promoter of astrocytes through TGF-β pathway [44]. Normalizaton of GFAP expression with low dose of ASH-WEX (0.1%) in glutamate (0.5 mM) treatment group depicted possible cytoprotective effect of the extract in C6 cells.

The expression of NF200 and its phosphorylated form was reduced upon treatment with glutamate as compared to control and corresponding ASH-WEX treatment groups. In an adult neuron, Neurofilaments (NFs) are major cytoskeletal components of neurons and are composed mainly of three different polypeptide subunits: NF-L (68 kDa); NF-M (160 kDa); and NF-H (200 kDa) [45]. Extensive phosphorylation of NFs at the carboxyterminal domains has been considered one of the means by which neurofilaments crosslink and stabilize the axonal cytoskeleton [46]. Therefore, NF200 degradation and dephosporylation in the glutamate treatment group may be a sign of loss of neuronal function and eventual neuronal cell death. The ASH-WEX treatment seems to overcome the glutamate induced adverse effects in IMR-32 cells with respect to NF200 expression and its phosphorylation proving its neuroprotective potential. Ashwagandha has been reported to protect against stress induced neuronal damage in rats due to its antioxidant properties [47]. Here we propose that the reversal of glutamate mediated changes in IFs could be partially attributed to its antioxidant mediated neuroprotective properties.

Glutamate exposure lead to increase in HSP70 expression in dose dependent manner which was reduced in low dose glutamate exposed cells treated with ASH-WEX. The HSP70 has been shown to have a neuroprotective role both in animal and cell culture models of neurotoxicity such as ischaemia [48], trauma [49], seizures [24] and Alzheimer's disease [50]. HSPs provide a line of defense against misfolded aggregation prone proteins and among the most potent suppressors of neurodegeneration in animal models [51]. Neurons may rely on their constitutive levels of HSC70 as a ‘pre-protection’ mechanism for defense against protein misfolding and aggregation that is induced by stressful stimuli or associated with neurodegenerative diseases. The expression of HSP70 was also upregulated in ASH-WEX alone treated group, thus suggesting that the ASH-WEX treatment could possibly induce HSP70 expression increasing protective capacity of cells against glutamate toxicity. In earlier studies, certain herbal extracts have been reported to induce HSP70 expression [52]. Our current results suggest that ASH-WEX treatment mediated induction of HSP70 expression may be one of the mechanisms for its neuroprotective potential against glutamate toxicity. The increase in expression of HSP70 and cell survival in the 1 mM glutamate exposed cells treated with ASH-WEX may be rescuing the cells under stress conditions. Overexpression of HSP70 has been reported to be associated with a decrease in apoptotic cell death and a reduction in matrix metalloproteinases [53].

In the present study, we further observed that MMP-2 and MMP-9 activity is upregulated during glutamate induced damage. Upon ASH-WEX treatment the expression was significantly lowered especially in the low dose glutamate treatment group in C6 and IMR-32 cells. Although MMP-2 is expressed constitutively in normal nerve cells, its expression is upregulated after injury. The temporal pattern of this activation coincides with nerve degeneration and suggests that MMP-2 plays a role in the regenerative process. Also, MMP-9 is detected in the nerve immediately following injury and is most abundant at the site of injury [54]. Another excitotoxic agent Kainic acid (KA) has been shown to induce neuronal degeneration by up regulation of MMPs expression. Similarly glutamate mediated upregulation of MMPs exacerbates neuronal and glial damage [55]. Consistently glutamate led to increase in MMPs expression in dose dependent manner. The exact mechanisms that trigger glutamate induced protease synthesis are not clear. It is evident in the present study that ASH-WEX intervention leads to protection of both the cell types, at least in low glutamate treated group, which may be explained by the decrease in expression of MMPs as shown by gelatin zymography.

NCAM and PSA-NCAM are important cell surface plasticity markers that play important role in regeneration and repair. NCAM is developmentally down-regulated but has been shown to increase after brain injury and this increase has been linked to potential of brain for regeneration [56]. In the present study, we observed marked increase in NCAM expression in glutamate treated group. ASH-WEX treatment further upregulated NCAM expression besides enhanced cell viability even at high dose of glutamate. Our study is consistent with the earlier report where excitotoxic increase in NCAM has been shown in hippocampal slices [57]. The increase in cell viability could be partially due to enhanced NCAM expression which is a potent neuroprotection conferring target as evident from previous studies [5], [56], [58]. Even soluble NCAM has been shown to interfere with glutamate-induced cell death in *in vitro* excitotoxicity assays. The growth factor, FGF-2 associated with NCAM signalling has been described to be neuroprotective against excitotoxicity caused by glutamate [59]. Control of PSA-NCAM expression by NMDA receptor activation has been described in several systems, suggesting a functional link between these two proteins. NMDA receptors exhibit a dichotomy of signalling with both toxic and plastic responses. Recent reports from our lab have shown that exposure to subtoxic concentration of NMDA results in a PSA-NCAM mediated neuroprotective state that was measured when these neurons were subsequently challenged with toxic doses of glutamate [31], [60]. Constituents of Withania have been associated with neuritic regeneration and synaptic reconstruction [3], [61]. NCAM and its polysialylated form being important molecules for CNS repair and regeneration, there may be direct association between NCAM and PSA-NCAM expression and ASH-WEX mediated regenerative and protective effects towards normalization and repair, which needs to be explored further.

PSA-NCAM expression was found to be significantly enhanced in response to glutamate induced excitotoxicity in C6 and IMR-32 cells, which may represent a compensatory mechanism to combat stress. In C6 cells, low dose glutamate exposure lead to significant increase in the PSA-NCAM expression level but percent change was less than the high dose treated cells. Moreover, the expression of PST in C6 cells showed dose dependent increase with increase in glutamate treatment groups and its expression was further elevated to significant level in the ASH-WEX treatment groups. In contrast, there was dose dependent increase in PSA-NCAM expression in glutamate treated IMR-32 cells. ASH-WEX treatment leads to further increase in expression of PSA-NCAM as well as PST. These differences could be possibly due to difference in cell type and therefore the differential expression of NCAM and degree of polysialylation on neuronal and glial cells.

Several studies have shown that PSA is a potent target to prevent excitotoxic neuronal cell death during development as well as under pathological conditions, resulting in glutamate release, at least in cases when glutamate is accumulated in the extracellular space at low concentrations. PSA has been proposed to inhibit activation of GluN2B-containing receptors, possibly by steric hindrance of the ligand to access the glutamate binding site at low micromolar concentrations of glutamate [62]. PSA has been shown to act as a neuroprotective agent, disconnecting overstimulated synapses to protect the relevant circuits from damage caused by excess glutamtergic input [63]. In another study the upregulation of HSP70 and PSA-NCAM by hyperthermia has been correlated and reported to significantly impact the hippocampal plasticity, permitting induction of the complex molecular cascade responsible for neuroprotection [64], [65]. In line with these results, it may be proposed that observations of increase in expression of HSP70 and PSA-NCAM upon glutamate treatment protected the cells from excitotoxic cell death. Pharmacological and biochemical analysis of PSA synthesis have suggested calcium dependent PST activity [66]. Thus the changes in the expression of PSA-NCAM in the present study may possibly be attributed to accumulation of intracellular calcium due to glutamate exposure. Alteration in PSA-NCAM expression levels on cell surface could also reflect differential delivery of PSA to cell surface as evident by a study in oligodendrocyte precursor cells in which NMDA induced influx of calcium probably enhanced transport of PSA to the cell surface [67]. PSA inhibits GluN2B-containing receptors at low micromolar concentrations of glutamate found in the extracellular space [68], [69]. It has been shown that PSA inhibits NMDAR currents at lower but not at higher concentrations of glutamate possibly by competing with glutamate in binding to positively charged amino acids. Furthermore, the expression and cleavage of the extracellular domain of NCAM/PSA-NCAM is regulated by metalloproteinase activity resulting in MMP induced proteolysis resulting in neuronal damage [70], [71]. Thus the decrease in MMP levels upon ASH-WEX treatment could possibly lead to cellular protection against any such damage. The increase in NCAM and PSA-NCAM expression upon glutamate exposure could be protective and regenerative response of the cells towards glutamate induced damage which is further enhanced by ASH-WEX treatment possibly leading to recovery of cells from excitotoxicity. In another study of glutamate-induced excitotoxicity it was revealed that treatment with PSA prevents cell death, whereas removal of neuronal cell surface-expressed PSA promotes cell death [72]. Thus, PSA carried by NCAM regulates both synaptic plasticity and viability via modulation of NMDA receptors. The increase in PSA levels seen in the current results may be functionally linked to cell tolerance e.g. protection against glutamate-induced cell death, which is apparent at lower concentration of glutamate only.

Withania extracts has been widely studied for their neuroprotective properties in animal models and *in vitro* studies. ASH-WEX comprises of six different water soluble molecules [38] which might be alone or in combination are associated with neuroprotective activity of the extract. One of the components of alcoholic extract of leaves, Withaonone has been shown to impart protection against Methoxyacetic acid (MAA) induced toxicity by suppressing the ROS levels, DNA and mitochondrial damage *in vitro* [73]. Its bioactive components Sitoindosides VII-X and withaferin A have been shown to modulate brain functions by binding with cholinergic receptors [74]. Modulation of release of three neurotransmitters i.e., acetylcholine, glutamate and serotonin by Withania in all probability contributes to inhibition of nNOS in extract treated stressed mice [40]. The neuroprotective properties of Withania have been attributed to neurochemical alterations of specific neurotransmitter systems and suppression of glucocorticoid release in chronic stress which could be exploited for treatment of neurodegenerative diseases [7]. Withania root extracts have been shown to impart protection against 6-hydroxydopamine induced rat model and various other animal models for neurological disorders [75], [76], [77]. Evidence also indicate that withanolide A, withanoside IV and withanoside VI from the Withania extract induced significant regeneration of both axons and dendrites, in addition to the reconstruction of pre- and postsynapses in the neurons [61]. The crude ethanolic extract of Withania roots has been shown to mitigate the effects of excitotoxicity and oxidative damage in hippocampus and the underlying mechanism could be attributed to its antioxidative properties [7], [47], [78]. Consistent with these neuroprotective properties of Withania extracts, present study illustrates the neuromodulatory role of aqueous extract from leaves of Withania against glutamate induced stress and upregulation of plasticity marker proteins such as HSP70, NCAM and PSA-NCAM may rescue the glial and neuronal cells from glutamate induced cytotoxicity.

The cytoprotective effects observed in this study could be attributed to the presence of free radical scavenging compounds in the water extract of Ashwagandha. In the present study low level glutamate induced effects were normalized by ASH-WEX but it could only partially revert the cytotoxic effects when challenged with high dose of glutamate. The higher expression of HSP70, NCAM and PSA-NCAM in response to glutamate exposure could be possibly due to cytoprotective response of cells towards excitotoxicity in the time frame of these experiments. ASH-WEX treatment lead to significant increase in viability in glutamate treated groups implicating its cytoprotective role against cytotoxicity. As elevated levels of glutamate have been implicated in a wide range of neurological diseases thus further research into the molecular mechanism of ASH-WEX mediated neuroprotection and the search for bioactive component(s) in these extracts may prove valuable therapeutic agent to combat neurological disorders.
